# Multi-gene phylogeny and taxonomy of the genus *Bannoa* with the addition of three new species from central China

**DOI:** 10.3389/fmicb.2023.1143156

**Published:** 2023-03-14

**Authors:** Chun-Yue Chai, Ting Lei, Xue-Ying Chu, Feng-Li Hui

**Affiliations:** ^1^School of Life Science and Agricultural Engineering, Nanyang Normal University, Nanyang, China; ^2^Research Center of Henan Provincial Agricultural Biomass Resource Engineering and Technology, Nanyang Normal University, Nanyang, China; ^3^School of Water Resources and Environment Engineering, Nanyang Normal University, Nanyang, China

**Keywords:** Basidiomycota, morphology, multigene analyzes, plant leaves, three new species

## Abstract

The genus *Bannoa* is a small group of ballistoconidium-forming yeasts in the family Erythrobasidiaceae (Cystobasidiomycetes). Prior to this study, seven species belonging to this genus have been described and published. In this study, phylogenetic analyzes of *Bannoa* based on the combined sequences of the small ribosomal subunit (SSU) rRNA gene, the internal transcribed spacer (ITS) regions, the D1/D2 domains of the large subunit rRNA gene (LSU) and the translation elongation factor 1-α gene (TEF1-α) were conducted. Three new species, namely *B. ellipsoidea*, *B. foliicola*, and *B. pseudofoliicola*, were delimited and proposed based on morphological and molecular evidence. *B. ellipsoidea* was found to be closely related to the type strains of *B. guamensis*, *B. hahajimensis*, and *B. tropicalis*, but with 0.7–0.9% divergence (4–5 substitutions) in the LSU D1/D2 domains and 3.7–4.1% divergence (19–23 substitutions and one−two gaps) in the ITS regions. *B. foliicola* was found to belong to the same clade as *B. pseudofoliicola* from which it differed by 0.4% divergence (two substitutions) in the LSU D1/D2 domains and 2.3% divergence (13 substitutions) in the ITS regions. The distinguishing morphological characteristics of the three new species, with respect to closely related taxa, are discussed. The identification of these new taxa significantly increases the number of *Bannoa* that have been described on the surface of plant leaves. Additionally, a key for the identification of *Bannoa* species is provided.

## Introduction

The genus *Bannoa*, belonging to Erythrobasidiales, is a kind of ballistoconidium-forming Basidiomycota. It was established by Hamamoto et al. (2002) based on a single species, *Bannoa hahajimensis*, which was isolated from dead leaves of plants collected in the Ogasawara Islands, Japan. Additionally, the anamorphic species obtained in this study were described as three new species of *Sporobolomyces*, namely *S*. *bischofiae*, *S*. *ogasawarensis*, and *S*. *syzygii*. However, these three species formed a monophyletic cluster with *Bannoa hahajimensis* in the *Erythobasidium* clade based on sequence analyzes of the SSU rRNA gene (Hamamoto et al., 2002), the D1/D2 domains of the LSU rRNA gene (Hamamoto, 2011), and a combined gene dataset of SSU rRNA, ITS regions, LSU rRNA, and first and second codon of TEF1-α (Nagahama et al., 2006). This monophyly was confirmed by Wang et al. et al. based on a seven-gene dataset consisting of SSU rRNA, D1/D2 LSU rRNA domains, ITS regions, RPB1, RPB2, TEF1-α and CYTB (Wang et al., 2015a). Recently, the genus *Bannoa* was emended by Wang et al. based on the phylogenetic analysis of a seven-gene dataset (Wang et al., 2015b). In this seven-gene phylogeny, the genus represented a well-supported clade containing a teleomorphic species *B. hahajimensis* and three *Sporobolomyces* species, including *S*. *bischofiae*, *S*. *ogasawarensis*, and *S*. *syzygii*, which were formerly recognized as the *Bannoa* clade (Hamamoto et al., 2002; Nagahama et al., 2006; Hamamoto, 2011; Wang et al., 2015a). Recent incorporations into the genus are *B*. *guamensis*, *B*. *rosea*, and *B*. *tropicalis* isolated from the surfaces of diseased and healthy leaves of plants in the Euphorbiaceae, Asteraceae, and Poaceae plant families (Parra and Aime, 2019). The genus *Bannoa* was located in the family Erythrobasidiaceae but was phylogenetically distinguished from other recognized species in the genus *Erythrobasidium* (Wang et al., 2015b).

Yeasts in the genus *Bannoa* are best known for their orange to salmon-red appearance in culture (Hamamoto, 2011; Wang et al., 2015b). The sexual morph of *Bannoa* is characterized by the production of unicellular basidia on a clamp connection formed after mating, while the asexual morph is characterized by budding cells, some of which are ballistoconidia that can be ovoidal or ellipsoidal (Hamamoto et al., 2002; Hamamoto, 2011; Parra and Aime, 2019).

The worldwide species diversity of *Bannoa* has not been extensively studied. Until now, only seven species have been accepted in the genus *Bannoa* (Wang et al., 2015b; Parra and Aime, 2019). Among them, *B*. *hahajimensis*, *B*. *ogasawarensis*, and *B*. *syzygii* are reported to occur in China and Japan (Zang et al., 1998; Chiang et al., 2001; Hamamoto et al., 2002; Li et al., 2020), while *B. bischofiae* has only been identified in Japan (Hamamoto et al., 2002), and *B. guamensis*, *B. rosea*, and *B. tropicalis* have been isolated in South America (Parra and Aime, 2019). In addition, several studies suggest the existence of other potentially new species that could belong to this genus (Matheny et al., 2006; Vaïtilingom et al., 2012; Wang et al., 2015a,b; Parra and Aime, 2019; Li et al., 2020).

During investigations of basidiomycetous yeasts from the Henan Province, central China, seven ballistoconidium-forming yeasts were collected. Morphological characteristics and phylogenetic analyzes based on SSU, ITS, LSU D1/D2, and TEF1-α sequences indicated that these yeasts represented three undescribed species of the *Bannoa* genus. This study confirms the taxonomic affinities of these new species, explores the species diversity of *Bannoa* in central China, and infers evolutionary relationships between representative species of *Bannoa*.

## Materials and methods

### Sample collection and yeast isolation

**Figure 1:**
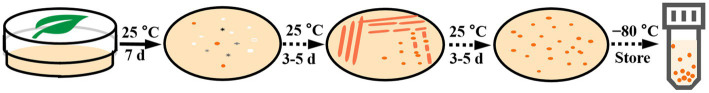
The yeast isolation procedures.

Plant leaves were collected from the Baotianman Nature Reserve (33°30′44″N, 111°55′47″E) in the Henan Province of central China. The protected area measures 4,285 ha and is classified as a world biosphere reserve by the United Nations Educational, Scientific and Cultural Organization (UNESCO). The local climate is a typical transitional climate spanning a northern subtropical zone to a warm temperate zone, with cold and dry winters and fresh and rainy summers, and with annual mean temperatures around 15°C (Hu et al., 2022). Plant leaf samples were stored in sterile flasks and transported under refrigerated conditions within 24 h of collection. Yeast strains were isolated from plant leaves by the ballistospore-fall method as previously described (Nakase and Takashima, 1993; Hu et al., 2022). The semi-withered leaf samples were stuck with vaseline to the inside of the lid of petri plates containing yeast malt (YM) agar (0.3% yeast extract, 0.3% malt extract, 0.5% peptone, 1% glucose, and 2% agar) and incubated at 25°C for 7 days, and the media was refreshed daily until colonies gradually formed. Different yeast morphotypes were picked and purified by streaking onto fresh YM agar. All purified yeast strains were suspended in YM broth supplemented with 20% (v/v) glycerol and stored at −80°C (Figure 1). Cultures of isolates obtained for this study were preserved in the Microbiology Lab, at Nanyang Normal University, Henan, China. All isolates used in this study and their origins are described in Table 1.

**Table 1:** Novel yeast strains isolated from plant leaves.

Strain	Source	Location
*B. ellipsoidea*		
NYUN 211090	Healthy undetermined leaf	Yingbishi, Baotianman Nature Reserve, Nanyang, Henan Province, China
NYUN 2110438	Healthy undetermined leaf	Baihualin, Baotianman Nature Reserve, Nanyang, Henan Province, China
NYUN 2110396^T^	Diseased leaf of *Dendrobenthamia* sp.	Baihualin, Baotianman Nature Reserve, Nanyang, Henan Province, China
*B. foliicola*		
NYNU 208237^T^	Healthy undetermined leaf	Yulianpu, Baotianman Nature Reserve, Nanyang, Henan Province, China
NYNU 219456	Puccinia-infected leaf of *Turpinia* sp.	Quancaizhuag, Baotianman Nature Reserve, Nanyang, Henan Province, China
*B. pseudofoliicola*		
NYUN 2110469^T^	Healthy leaf of *Impatiens* sp.	Baihualin, Baotianman Nature Reserve, Nanyang, Henan Province, China
NYUN 2106285	Healthy leaf of *Impatiens* sp.	Baihualin, Baotianman Nature Reserve, Nanyang, Henan Province, China

### Morphological and physiological characterization

Morphological and physiological traits of the yeast strains were characterized according to methods established by Kurtzman et al. (2011). Colony traits were observed on YM agar after 2 weeks of incubation at 25°C. Mycelium formation was investigated by cultivation on corn meal (CM) agar (2% cornmeal infusion and 2% agar) in slide culture at 25°C for 2 weeks. The sexual processes of all strains were examined for individual strains and strain pairs on potato dextrose agar (PDA) (20% potato infusion, 2% glucose, and 1.5% agar), CM agar, and yeast carbon base plus 0.01% ammonium sulfate (YCBS) agar at 25°C for 2 months (Hamamoto et al., 2002; Parra and Aime, 2019). Glucose fermentation was carried out in a liquid medium using Durham fermentation tubes. Carbon and nitrogen source assimilation tests were conducted in liquid medium and starved inocula were used for the nitrogen assimilation tests (Kurtzman et al., 2011). Cycloheximide resistance tests were also performed in a liquid medium, while urea hydrolysis assays were conducted on agar slants. Acid production and reaction with diazonium blue B (DBB) were investigated on a solid medium in petri dishes. Growth at various temperatures (15, 20, 25, 30, 35, and 37°C) was determined by cultivation on YM agar. Cell morphology was examined using a Leica DM 2500 microscope (Leica Microsystems GmbH, Wetzlar, Germany) with a Leica DFC295 digital microscope color camera using bright field, phase contrast, and differential interference contrast (DIC) optics. Novel taxonomic descriptions and proposed names were deposited in MycoBank (http://www.mycobank.org; 8 January 2023).

### DNA extraction, PCR amplification, and sequencing

Genomic DNA was extracted from the yeasts using the Ezup Column Yeast Genomic DNA Purification Kit according to the manufacturer’s protocol (Sangon Biotech, China). Briefly, yeast cells were homogenized in snailase reaction buffe, followed by using with solution to subsequently remove protein. The resulting DNA was further purified by adsorption column and resuspended in 50 μL TE Buffer. A total of four nuclear loci were sequenced, including the small ribosomal subunit (SSU) rRNA gene, the internal transcribed spacer (ITS) regions, the D1/D2 domains of the large subunit (LSU) rRNA gene, and the translation elongation factor 1-α gene (TEF1-α). The primer pairs NS1/NS8 (White et al., 1990), ITS1/ITS4 (White et al., 1990), NL1/NL4 (Kurtzman and Robnett, 1998), and EF1-526F/EF1-1567R (Rehner and Buckley, 2005) were used for amplifying SSU, ITS, LSU, andTEF1-α, respectively.

PCR was conducted as described by Toome et al. (2013) for SSU, ITS, and LSU. For TEF1-α, a touchdown PCR protocol was used as described by the research group (Wang et al., 2014). PCR products were directly purified and sequenced by Sangon Biotech Inc. (Shanghai, China). We determined the identity and accuracy of the newly obtained sequences by comparing them to sequences in GenBank and assembled them using BioEdit (Hall, 1999). Newly obtained sequences were then submitted to GenBank (https://www.ncbi.nlm.nih.gov/genbank/; Table 2).

**Table 2:** Sequences used for the molecular phylogenetic analysis Entries in bold were newly generated in this study.

Species Name	Srains no.	Locality	GenBank accession no.			
			SSU	ITS	LSU D1/D2	TEF1-α
*Bannoa bischofiae*	JCM 10338^T^	Japan	AB035721	AB035721	NG_058609	AB127094
***B. ellipsoidea***	**NYUN 211090**	**China**	**OP218260**	**OP221007**	**OP221005**	**OP750515**
***B. ellipsoidea***	**NYUN 2110438**	**China**	**OP221004**	**OP221008**	**OP221017**	**OP750516**
***B. ellipsoidea***	**NYUN 2110396** ^**T**^	**China**	**OP221010**	**OM014197**	**OM014195**	**OP725922**
***B. foliicola***	**NYNU 208237** ^**T**^	**China**	**OP218261**	**MW365541**	**MW365544**	**OP750517**
***B. foliicola***	**NYNU 219456**	**China**	**OP221012**	**OP221021**	**OP221002**	**OP776181**
*B. guamensis*	CBS 16127^T^	United States	MK254996	MK287350	MK255006	MK491345
*B. hahajimensis*	JCM 10336^T^	Japan	AB035897	AB035897	NG_042311	KJ707750
*B. ogasawarensis*	JCM 10330^T^	Japan	AB035717	AB035717	NG_058699	AB127095
*B. rosea*	CBS 16128^T^	United States	–	MK287351	MK255007	MK491346
*B. syzygii*	JCM 10337^T^	Japan	AB035720	AB035720	NG_058700	AB127096
*B. tropicalis*	CBS 16087^T^	United States	MK255003	MK287360	MK255016	MK491353
***B. pseudofoliicola***	**NYUN 2110469** ^**T**^	**China**	**OP221018**	**OM014200**	**OM014198**	**OP750518**
***B. pseudofoliicola***	**NYUN 2106285**	**China**	**OP218264**	**OP221006**	**OP221009**	**OP776180**
*Cyrenella elegans*	CBS 274.8^T^	United States	NG_061174	NR_145383	NG_058875	KJ707830
*Erythrobasidium elongatum*	CBS 8080^T^	Australia	NG_063449	NR_073306	NG_059254	AB127099
*E. hasegawianum*	JCM 1545^T^	United States	D12803	NR_111008	AF131058	KJ707776
*E. yunnanense*	CBS 8906^T^	China	NG_063520	NR_155098	NG_059190	AB127100
*Hasegawazyma lactosa*	CBS 5826^T^	Japan	D45366	NR_073295	NG_057668	AB127098
*Naohidea sebacea*	CBS 8477^T^	–	KP216515	NR_121324	NG_042442	KJ707783

CBS, CBS-KNAW Collections, Westerdijk Fungal Biodiversity Institute, Utrecht, The Netherlands; JCM, Japan Collection of Microorganisms, RIKEN BioResource Research Center, Tsukuba, Japan; NYNU, Microbiology Lab, Nanyang Normal University, Henan, China; T, type strain.

### Phylogenetic analysis

The phylogenetic relationship of the new *Bannoa* species and related species was determined by analysis of concatenated sequence datasets of four loci (SSU, ITS, LSU D1/D2, and TEF1-α). *Naohidea sebacea* CBS 8477 was used as the outgroup, according to Wang et al. (2015a). For the dataset, each gene region was aligned using MAFFT v7.110 with default settings (Katoh and Standley, 2013), and then manually adjusted in BioEdit 7.1.3.0 (Hall, 1999). The“Concatenate Sequence” function in PhyloSuite v1.16 was used to combine the aligned sequences of the different loci (Zhang et al., 2020). Manual gap adjustments were done to improve the alignment and ambiguously aligned regions were also excluded. Multi-locus phylogenetic analyzes were performed with MEGA11 software (Tamura et al., 2021) using the neighbor-joining (NJ) and maximum likelihood (ML) methods. The Kimura-2 parameter distance correction (Saitou and Nei, 1987) and the general time reversible (GTR) models (Nei and Kumar, 2000) were, respectively, used for the NJ and ML analyzes. Confidence levels of the clades were estimated from bootstrap analysis (1,000 replicates) (Felsenstein, 1985).

## Results

### Molecular phylogenetic analysis

**Figure 2:**
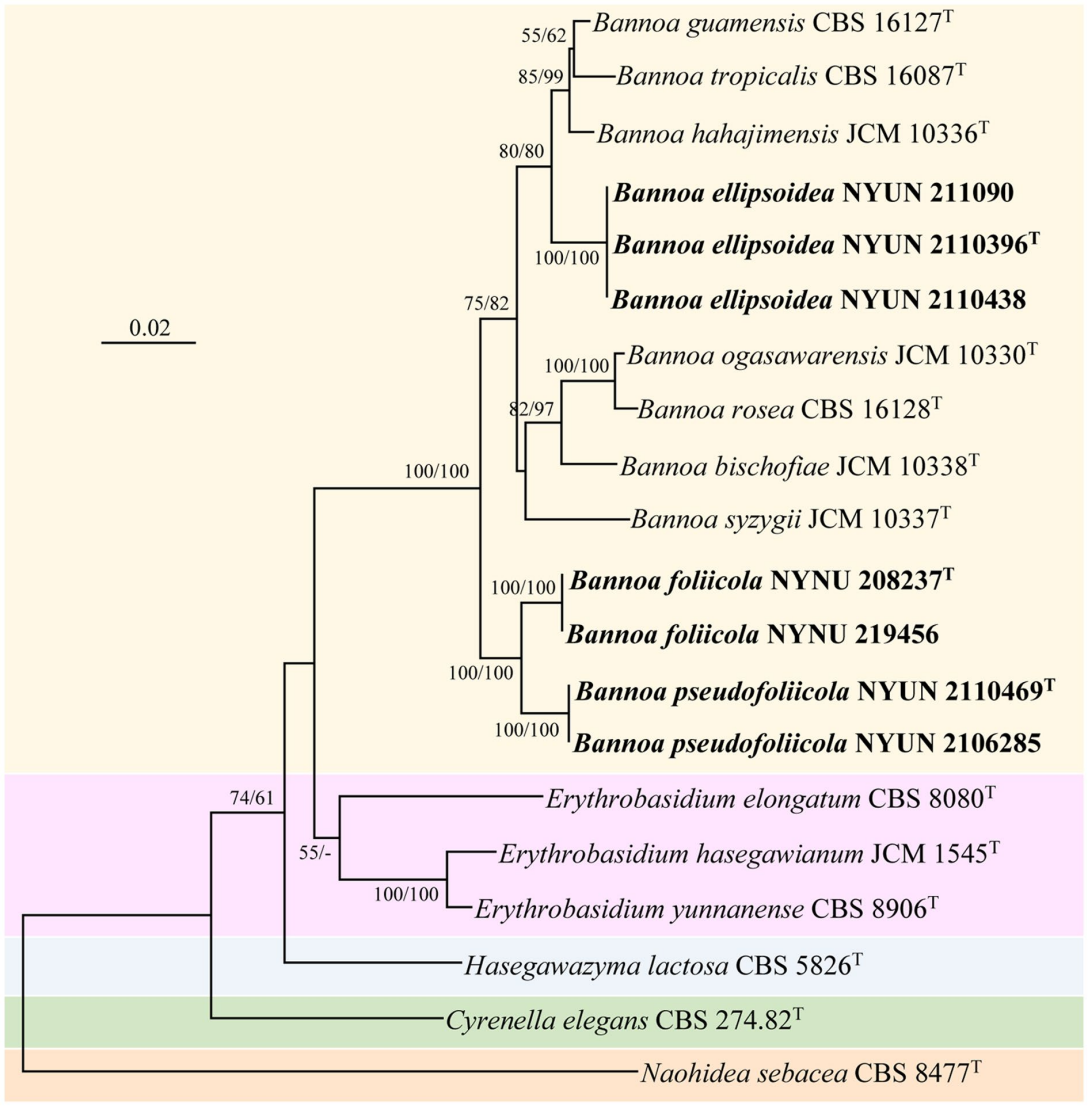
Maximum likelihood (ML) phylogram of *Bannoa* species based on the combined SSU + ITS + LSU D1/D2 + *tef1* sequence dataset. *Naohidea sebacea* CBS 8477^T^ was used as the outgroup. ML and neighbor-joining (NJ) bootstrap support values above 50% are shown at the nodes (ML/NJ). The novel strains described in this study are shown in bold.

The combined SSU + ITS + LSU D1/D2 + TEF1-α dataset was analyzed to infer the taxonomic positions of the novel species described in this study. The dataset included sequences from 20 fungal samples representing 16 taxa including the outgroup, *Naohidea sebacea* CBS 8477^T^. After alignment and trimming, the combined dataset contained 3,075 characters including gaps with 1,396 characters for SSU, 428 characters for ITS, 504 characters for LSU, and 747 characters for TEF1-α alignment, of which 358 characters were parsimony-informative. The best model for the SSU + ITS + LSU D1/D2 + TEF1-α dataset estimated and applied in the ML analysis was GTR + I + G with equal frequency of nucleotides. ML and NJ analyzes produced almost identical topologies, thus only the ML generated tree is displayed with ML and NJ supported values above 50% at the nodes (Figure 2). Our phylogenetic analyzes indicated that *Bannoa* formed a monophyletic group with high support (100% ML; 100% NJ; Figure 2). Ten phylogenetic species of the genus *Bannoa* are delineated, three of which were new: *B. ellipsoidea*, *B. foliicola*, and *B. pseudofoliicola*.

*Bannoa ellipsoidea* was grouped with the three described species, *B. guamensis, B. hahajimensis*, and *B. tropicalis* (Hamamoto et al., 2002; Parra and Aime, 2019), with strong support (80% ML; 80% NJ; Figure 2). The LSU D1/D2 sequences of *B. ellipsoidea* differed by 0.7% divergence (4 substitutions) from the type strain of *B. tropicalis*, and by 0.9% divergence (5 substitutions) from the type strains of *B. guamensis* and *B. hahajimensis*. Surprisingly, significant sequence differences between *B. ellipsoidea* and its close relatives, *B. guamensis, B. hahajimensis*, and *B. tropicalis*, were observed in the ITS regions. In these regions, *B. ellipsoidea* differed from the type strain of *B. tropicalis* by 3.7% divergence (19 substitutions and one gaps). Similarly, *B. ellipsoidea* exhibited 3.7% divergence (21 substitutions and two gaps) from the type strain of *B. guamensis* and 4.1% divergence (23 substitutions and one gap) from the type strain of *B. hahajimensis* in the ITS regions. According to the basidiomycetous yeast species thresholds proposed by Fell et al. (2000), Scorzetti et al. (2002), and Vu et al. (2016), strains that differ by two or more nucleotide substitutions in the D1/D2 domains or 1–2% nucleotide differences in the ITS regions may represent different taxa. The differences in both the LSU D1/D2 and ITS sequences were significant enough for *B. ellipsoidea* to be considered a distinct *Bannoa* species.

*Bannoa foliicola* formed a subclade with *B. pseudofoliicola* described in this study with full support (100% ML; 100% NJ; Figure 2). *B. foliicola* differed from its nearest phylogenetic neighbor, *B. pseudofoliicola* CBS 6936^T^, by 0.4% divergence (2 substitutions) in the LSU D1/D2 domains and 2.3% divergence (13 substitutions) in the ITS regions. According to the criteria mentioned above, this data clearly supports the distinction between *B. foliicola* and *B. pseudofoliicola* at the species level.

### Phenotypic characterization

**Figure 3:**
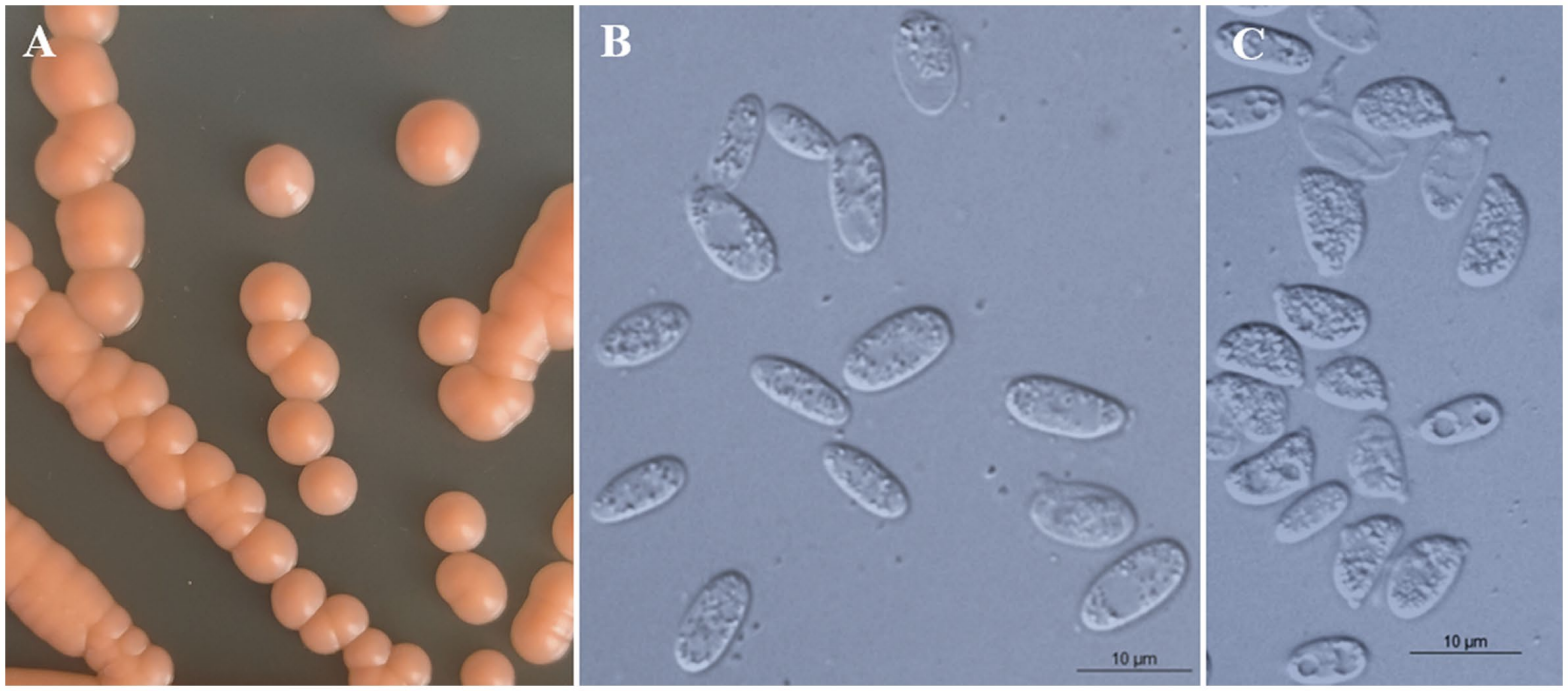
Morphological characteristics of *Bannoa ellipsoidea* sp. nov. (GDMCC 2.279, holotype). **(A)** Colony morphology on YM agar after growth for 7 d at 25°C. **(B)** Budding cells after growth for 7 d in YM broth at 25°C. **(C)** Ballistoconidia on CM agar after growth for 7 d at 25°C. Scale bars = 10 μm.

**Figure 4:**
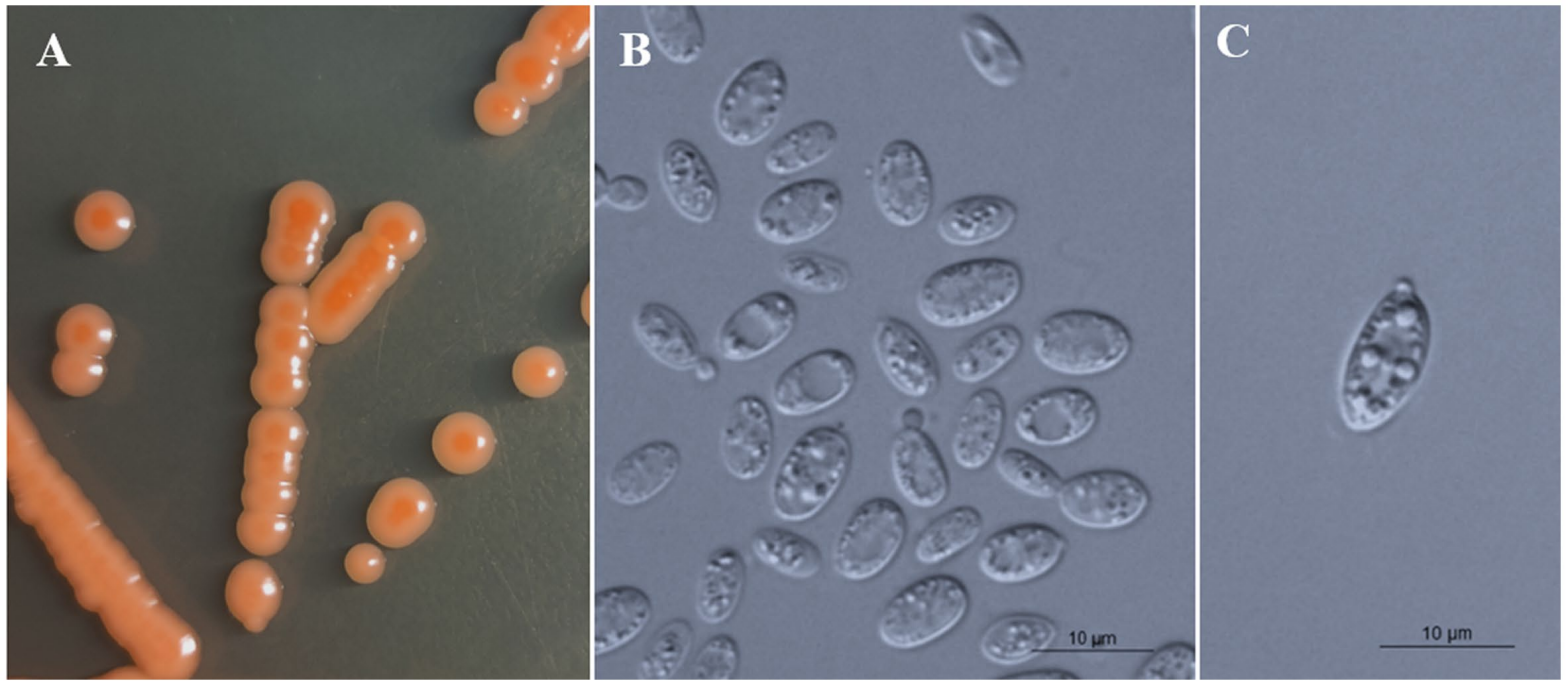
Morphological characteristics of *Bannoa foliicola* sp. nov. (CICC 33507, holotype). **(A)** Colony morphology on YM agar after growth for 7 d at 25°C. **(B)** Budding cells after growth for 7 d in YM broth at 25°C. **(C)** Ballistoconidia on CM agar after growth for 7 d at 25°C. Scale bars = 10 μm.

**Figure 5:**
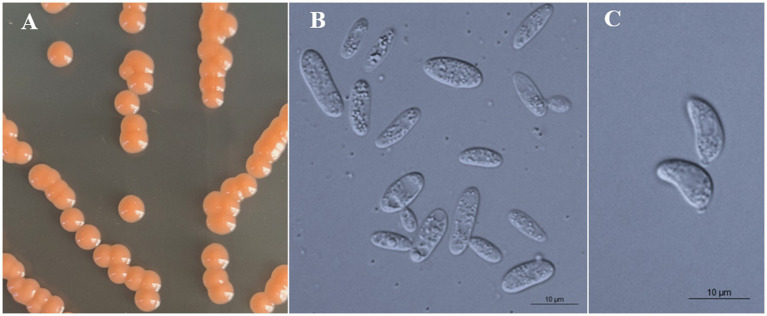
Morphological characteristics of *Bannoa pseudofoliicola* sp. nov. (GDMCC 2.270, holotype). **(A)** Colony morphology on YM agar after growth for 7 d at 25°C. **(B)** Budding cells after growth for 7 d in YM broth at 25°C. **(C)** Ballistoconidia on CM agar after growth for 7 d at 25°C. Scale bars = 10 μm.

All strains of the three new species formed orange-colored colonies (Figures 3A, 4A, 5A) and ovoidal, ellipsoidal, and cylindrical vegetative cells (Figures 3B, 4B, 5B) like other *Bannoa* species. Ballistoconidia were formed and were ovoidal and ellipsoidal in shape (Figures 3C, 4C, 5C). *B. ellipsoidea* produced more abundant ballistoconidia than strains of the other two new species. Sexual structures were not observed in the cultures of single or mixed strains on PDA, CM agar, or YCBS agar.

Phenotypic characteristics that differed between *B. ellipsoidea*, *B. foliicola,* and *B. pseudofoliicola* and their closely related species in the genus *Bannoa* are described in Table 3. *B. ellipsoidea* physiologically differed from *B. guamensis, B. hahajimensis*, and *B. tropicalis* in its inability to utilize L-arabinose, sucrose, maltose, melibiose, and D-mannitol (Table 3). Similarly, *B. foliicola* could be differentiated from *B. pseudofoliicola* by its ability to assimilate L-arabinose, D-arabinose, L-rhamnose, lactose, erythritol, galactitol, DL-lactate, succinate, and creatine and its inability to assimilate salicin. In addition, *B. foliicola* could grow at 35°C, while *B. pseudofoliicola* could not.

**Table 3:** Phenotypic characteristics that differed between the three novel species and their most closely related taxa.

Characteristics	1	2	3	4^*^	5^*^	6^*^
*Carbon assimilation*						
L-Sorbose	+	w	−	w	−	+
D-Glucosamine	+	w	−	w	−	n
D-Ribose	+	w	−	n	n	v
L-Arabinose	+	−	−	w	w	+
D-Arabinose	+	−	−	w	w	v
L-Rhamnose	+	−	w	w	w	+
Sucrose	+	w	−	w	w	+
Maltose	w	+	−	w	w	+
Methyl-α-D-glucoside	+	+	−	n	n	+
Cellobiose	+	w	−	+	w	+
Salicin	−	w	w	w	w	+
Arbutin	−	w	w	−	w	n
Melibiose	+	w	−	w	w	+
Lactose	+	−	w	−	d	−
Melezitose	+	+	−	w	w	+
Erythritol	+	−	−	n	n	−
Ribitol	w	w	−	n	n	v
D-Glucitol	+	w	−	n	n	+
D-Mannitol	w	+	−	w	w	+
Galactitol	w	−	−	d	−	v
D-Gluconate	+	+	−	w	+	n
DL-Lactate	+	−	−	d	d	v
Succinate	+	−	−	−	−	+
Methanol	−	−	−	−	d	−
Ethanol	−	−	−	d	d	v
*Nitrogen assimilation*						
Cadaverine	−	−	+	w	−	−
Creatine	w	−	−	w	−	n
Growth tests						
Growth at 35°C	w	−	−	−	−	+

Species: 1, *B. foliicola*; 2, *B. pseudofoliicola*; 3, *B. ellipsoidea*; 4, *B. tropicalis*; 5, *B. guamensis*; 6, *B. hahajimensis*. +, positive reaction; −, negative reaction; w, weakly positive; v, variable reaction; n, data not available. All data from this study, except ^*^ which were obtained from the original description (Hamamoto, 2011; Parra and Aime, 2019).

### Taxonomy

*Bannoa ellipsoidea* C.Y. Chai & F.L. Hui, sp. nov., Figure 3.

MycoBank: MB 847130.

Etymology: the specific epithet “*ellipsoidea*” refers to the ellipsoidal vegetative cells of the type strain.

Typus: China, Henan Province, Neixiang County, Baotianman Nature Reserve, in phylloplane from diseased leaf of *Dendrobenthamia* sp., October 2021, L. Zhang and H. zhang, NYUN 2110396 (holotype GDMCC 2.279^T^ preserved as a metabolically inactive state, culture ex-type JCM 35734).

Description: On YM agar, after 7 d at 25°C, colonies are orange, smooth, glistening and butyrous in texture (Figure 3A). The margin is entire. In YM broth, after 7 d at 25°C, cells are ellipsoidal and cylindrical, 3.2–4.1 × 8.3–9.5 μm and single, budding is polar (Figure 3B). After 1 mo at 25°C, a ring and sediment are present. In Dalmau plate culture on corn meal agar, pseudohyphae are not formed. Sexual structures are not observed on PDA, CM agar and YCBS agar. Ballistoconidia are ovoid and ellipsoidal, 5.0–6.3 × 7.4–13.3 μm (Figure 3C). Glucose fermentation is absent. Glucose, inulin, raffinose (weak), galactose (weak), lactose (weak), trehalose (weak), salicin (weak), L-rhamnose (weak), D-xylose (weak) and glycerol (weak) are assimilated as sole carbon. Sucrose, melibiose, maltose, melezitose, methyl-α-D-glucoside, cellobiose, L-sorbose, L-arabinose, D-arabinose, D-ribose, methanol, ethanol, erythritol, ribitol, galactitol, D-mannitol, D-glucitol, myo-inositol, DL-lactate, succinate, citrate, D-gluconate, D-glucosamine, D-glucono-1,5-lactone, 2-keto-D-gluconate, 5-keto-D-gluconate and D-glucuronate are not assimilated. Cadaverine is assimilated as sole nitrogen sources. Nitrate, nitrite, ethylamine, L-lysine, creatine, creatinine, glucosamine, imidazole and D-tryptophan are not assimilated. Maximum growth temperature is 30°C. Starch-like substances are not produced. Urease activity is positive. Diazonium Blue B reaction is positive.

Additional strain examined: China, Henan Province, Neixiang County, Baotianman Nature Reserve, in phylloplane from healthy undetermined leaf, October 2021, L. Zhang and H. zhang, NYUN 211090 and NYUN 2110438.

GenBank accession numbers: holotype GDMCC 2.279^T^ (SSU: OP221010, ITS: OM014197, D1/D2: OM014195, TEF1-α: OP725922); additional strains NYUN 211090 (SSU: OP218260, ITS: OP221007, D1/D2: OP221005, TEF1-α: OP750515) and NYUN 2110438 (SSU: OP221004, ITS: OP221008, D1/D2: OP221017, TEF1-α: OP750516).

*Bannoa foliicola* C.Y. Chai & F.L. Hui, sp. nov., Figure 4.

MycoBank: MB 847131.

Etymology: the specific epithet “*foliicola*” refers to the substrate origin of the type strain, leaves.

Typus: China, Henan Province, Neixiang County, Baotianman Nature Reserve, in phylloplane from healthy undetermined leaf, July 2016, L. Zhang and H. zhang, NYNU 208237 (holotype CICC 33507^T^ preserved as a metabolically inactive state, culture ex-type CBS 16656).

Description: On YM agar, after 7 d at 25°C, colonies are orange to salmon-red, smooth, glistening and butyrous in texture (Figure 4A). The margin is entire. In YM broth, after 7 d at 25°C, cells are ovoid and ellipsoidal, 2.1–4.4 × 6.4–8.0 μm and single, budding is polar (Figure 4B). After 1 mo at 25°C, a ring and sediment are present. In Dalmau plate culture on corn meal agar, pseudohyphae are not formed. Sexual structures are not observed on PDA, CM agar and YCBS agar. Ballistoconidia are ovoid and ellipsoidal, 5.2–6.9 × 10.8–14.3 μm (Figure 4C). Glucose fermentation is absent. Glucose, inulin, sucrose, raffinose, melibiose, galactose (weak), lactose, trehalose, maltose (weak), melezitose, methyl-α-D-glucoside, cellobiose, L-sorbose, L-rhamnose, D-xylose, L-arabinose, D-arabinose, D-ribose, glycerol, erythritol, ribitol (weak), galactitol (weak), D-mannitol (weak), D-glucitol, DL-lactate, succinate, D-gluconate, D-glucosamine, 2-keto-D-gluconate and D-glucuronate are assimilated as sole carbon sources. Salicin, methanol, ethanol, myo-inositol, citrate, D-glucono-1,5-lactone and 5-Keto-D-gluconate are not assimilated. L-Lysine, creatine (weak), glucosamine and D-tryptophan are assimilated as sole nitrogen sources. Nitrate, nitrite, ethylamine, cadaverine, creatinine and imidazole are not assimilated. Maximum growth temperature is 35°C. Growth in vitamin-free medium is positive. Starch-like substances are not produced. Urease activity is positive. Diazonium Blue B reaction is positive.

Additional strain examined: China, Henan Province, Neixiang County, Baotianman Nature Reserve, in phylloplane from puccinia-infected leaf of *Turpinia* sp., October 2021, L. Zhang and H. zhang, NYNU 219456.

GenBank accession numbers: holotype CICC 33507^T^ (SSU: OP218261, ITS: MW365541, D1/D2: MW365544, TEF1-α: OP750517); additional strain NYNU 219456 (SSU: OP221012, ITS: OP221021, D1/D2: OP221002, TEF1-α: OP776181).

*Bannoa pseudofoliicola* C.Y. Chai & F.L. Hui, sp. nov., Figure 5.

MycoBank: MB 847132.

Etymology: the specific epithet “*pseudofoliicola*” refers to the similar colony morphology to that of *Bannoa foliicola*.

Typus: China, Henan Province, Neixiang County, Baotianman Nature Reserve, in phylloplane from healthy undetermined leaf, October 2021, L. Zhang and H. zhang, NYNU 2110469 (holotype GDMCC 2.270^T^ preserved as a metabolically inactive state, culture ex-type JCM 35726).

Description: On YM agar, after 7 d at 25°C, colonies are orange, smooth, glistening and butyrous in texture (Figure 5A). The margin is entire. In YM broth, after 7 d at 25°C, cells are ellipsoidal and cylindrical, 3.2–3.7 × 7.7–12.6 μm and single, budding is polar (Figure 5B). After 1 mo at 25°C, a pellicle and sediment are present. In Dalmau plate culture on corn meal agar, pseudohyphae are not formed. Sexual structures are not observed on PDA, CM agar and YCBS agar. Ballistoconidia are ovoid and ellipsoidal, 4.7–7.0 × 9.0–11.4 μm (Figure 5C). Glucose fermentation is absent. Glucose, inulin (weak), sucrose (weak), raffinose (weak), melibiose (weak), galactose (weak), trehalose, maltose, melezitose, methyl-α-D-glucoside, cellobiose (weak), salicin (weak), L-sorbose (weak), D-xylose (weak), D-ribose (weak), glycerol (weak), ribitol (weak), D-mannitol, D-glucitol (weak), D-gluconate, D-glucosamine (weak), 2-keto-D-gluconate and D-glucuronate are assimilated as sole carbon sources. Lactose, L-rhamnose, L-arabinose, D-arabinose, methanol, ethanol, erythritol, galactitol, myo-inositol, DL-lactate, succinate, citrate, D-glucono-1,5-lactone and 5-keto-D-gluconate are not assimilated. Creatinine (weak), glucosamine (weak) and D-tryptophan (weak) are assimilated as sole nitrogen sources. Nitrate, nitrite, ethylamine, L-lysine, cadaverine, creatine and imidazole are not assimilated. Maximum growth temperature is 30°C. Growth in vitamin-free medium is positive. Starch-like substances are not produced. Urease activity is positive. Diazonium Blue B reaction is positive.

Additional strain examined: China, Henan Province, Neixiang County, Baotianman Nature Reserve, in phylloplane from healthy undetermined leaf, October 2021, L. Zhang and H. zhang, NYUN 2106285.

GenBank accession numbers: holotype GDMCC 2.270^T^ (SSU: OP221018, ITS: OM014200, D1/D2: OM014198, TEF1-α: OP750518); additional strain NYUN 2106285 (SSU: OP218264, ITS: OP221006, D1/D2: OP221009, TEF1-α: OP776180).

### Key to species of *Bannoa*

The main trait comparisons and key identification of *Bannoa* are summarized below for the reader’s convenience (Table 4).

**Table:** 

1. a. Melezitose is assimilated	2
b. Melezitose is not assimilated	*B. ellipsoidea*
2. (1) a. Inulin is assimilated	3
b. Inulin is not assimilated	7
3. (2) a. Glycerol is assimilated	4
b. Glycerol is not assimilated	*B. rosea*
4. (3) a. L-Rhamnose is assimilated	5
b. L-Rhamnose is not assimilated	*B. pseudofoliicola*
5. (4) a. L-Sorbose is assimilated	6
b. L-Sorbose is not assimilated	*B. guamensis*
6. (5) a. Salicin is assimilated	*B. tropicalis*
b. Salicin is not assimilated	*B. foliicola*
7. (2) a. Growth at 30°C	8
b. Growth is absent at 30°C	*B. ogasawarensis*
8. (7) a. L-Arabinose is assimilated	9
b. L-Arabinose is not assimilated	*B. bischofiae*
9. (8) a. Growth at 35°C	*B. hahajimensis*
b. Growth is absent at 35°C	*B. syzygii*

**Table 4:** The main characteristics of species assigned to the genus *Bannoa*.

Species Name	Growth in/at								
	L-Sorbose	L-Arabinose	L-Rhamnose	Melezitose	Inulin	Glycerol	Salicin	30°C	35°C
*B. rosea*	w	w	w	w	w	−	−	+	−
*B. ogasawarensis*	+	+	v	+	−	+	+	−	−
*B. foliicola*	+	+	+	+	+	+	−	+	w
*B. pseudofoliicola*	w	−	−	+	w	w	w	+	−
*B. ellipsoidea*	−	−	w	−	+	w	w	+	−
*B. tropicalis*	w	w	w	w	+	w	w	+	−
*B. guamensis*	−	w	w	w	+	w	w	+	−
*B. bischofiae*	+	−	n	+	−	+	+	+	+
*B. hahajimensis*	+	+	+	+	−	+	+	n	+
*B. syzygii*	+	+	n	+	−	+	+	+	−

+, positive reaction; −, negative reaction; w, weakly positive; v, variable reaction; n, data not available.

## Discussion

In this study, three species of *Bannoa* from China, *B. ellipsoidea*, *B. foliicola*, and *B. pseudofoliicola*, are described as new species based on molecular analyzes and morphological features. Phylogenetic analyzes of these three new species of *Bannoa* confirmed that *Bannoa* is a monophyletic genus in a strongly supported clade that was similar to those described by Wang et al. (2015a,b) and Parra and Aime (2019). These three new species were clearly separated from other *Bannoa* species and from each other (Figure 2). Pairwise sequence comparisons of the LSU D1/D2 domains and the ITS regions between these three species and related species showed that they had lower similarity values than the common threshold for species demarcation in basidiomycetous yeast (Fell et al., 2000; Scorzetti et al., 2002; Vu et al., 2016). Additionally, they were highly similar in colony morphology and color, as well as in cell shape, but differed markedly from the closest related species in terms of their physiological and biochemical characteristics (Table 3). Phylogenetic analyzes coupled with morphological studies confirm the existence of these species in China.

Most reported *Bannoa* isolates were isolated from dead asymptomatic or infected leaves. *B. bischofiae*, *B. hahajimensis*, *B. ogasawarensis,* and *B. syzygii* were isolated from dead leaves of various plant species in the Ogasawara, Iriomote-Jima, and Yakushima islands of subtropical southwestern Japan (Hamamoto et al., 2002), or from the surface of leaves of *Miscanthus* and other unidentified plants in China (Zang et al., 1998; Chiang et al., 2001; Li et al., 2020). *B. guamensis*, *B. rosea*, and *B. tropicalis* were isolated from surfaces of diseased and healthy leaves of plants in the Euphorbiaceae, Asteraceae, and Poaceae plant families from the South Pacific Island of Guam and Guyana in South America (Parra and Aime, 2019). In addition, two strains of *Bannoa,* isolated from teliospores of *Cintractia axicola* in Panama (Matheny et al., 2006) and from cloud water samplings in France (Vaïtilingom et al., 2012), have been identified but not yet been named or extensively described (Parra and Aime, 2019). In the current study, we describe three new species of the *Bannoa* genus, *B. ellipsoidea*, *B. foliicola* and *B. pseudofoliicola*, isolated from healthy or diseased leaves collected in subtropical central China. Based on the evidence provided above, it is suggested that the phylloplane of plant leaves is the main habitat of yeasts in the genus *Bannoa*.

Members of *Bannoa* have not been sufficiently studied, and the diversity of *Bannoa* is poorly understood. All known *Bannoa* species, including *B. ellipsoidea*, *B. foliicola* and *B. pseudofoliicola* described in the present study, have a relatively narrow geographic distribution range, found primarily in subtropical Asia and tropics South America. However, some strains of *Bannoa* have been isolated in different parts of the world; for example, *Bannoa* sp. MP3490 (DQ631900) has been obtained from Panama, *Bannoa* aff. *Ogasawarensis* MCA7670 (MK990652) and *Bannoa* aff. *Ogasawarensis* MCA 7643 (MK990651) from Vanuatu, *Bannoa* aff. *Guamensis* MCA7799 (MK990655) from Cameroon, and *Bannoa* sp. BRIP 28272 (OK001795) from Australia. In addition, several environmental sequences of *Bannoa* have also been reported from Mexico (James et al., 2016), from Austria, and from Australia (Raghavendra et al., 2017). This suggests this genus could be broadly distributed and further large-scale studies are needed to explore the diversity and distribution of *Bannoa* species worldwide. Ultimately, these findings will greatly improve our understanding of the diversity, distribution and ecology of *Bannoa*.

In conclusion, three new *Bannoa* species isolated from the surface of plant leaves in China are identified based on morphology and phylogeny; *viz. B. ellipsoidea*, *B. foliicola* and *B. pseudofoliicola*. This study enriches the species diversity of the genus, which will also promote its taxonomy and phylogeny.

## Data availability statement

The datasets presented in this study can be found in online repositories. The names of the repository/repositories and accession number(s) can be found in the article/Supplementary material.

## Author contributions

C-YC and X-YC isolated the yeast strains and performed the taxonomic characterization. TL performed the phylogenetic analysis. C-YC drafted the manuscript and prepared the tables and figures. F-LH designed the study and revised the manuscript. All authors contributed to the article and approved the submitted version.

## Funding

This research was funded by the National Natural Science Foundation of China (Project no. 31570021), Key scientific research projects of colleges and universities in Henan Province China (Project no. 23A210018) and the State Key Laboratory of Motor Vehicle Biofuel Technology, Henan Tianguan Enterprise Group Co., Ltd., China (no. 2018001).

## Conflict of interest

The authors declare that the research was conducted in the absence of any commercial or financial relationships that could be construed as a potential conflict of interest.

## Publisher’s note

All claims expressed in this article are solely those of the authors and do not necessarily represent those of their affiliated organizations, or those of the publisher, the editors and the reviewers. Any product that may be evaluated in this article, or claim that may be made by its manufacturer, is not guaranteed or endorsed by the publisher.
